# USP10 promotes pancreatic ductal adenocarcinoma progression by attenuating FOXC1 protein degradation to activate the WNT signaling pathway

**DOI:** 10.7150/ijbs.92278

**Published:** 2024-09-30

**Authors:** Jie Wang, Lang Gan, Fenghao Liu, Qin Yang, Qingsong Deng, Di Jiang, Chengcheng Zhang, LeiDa Zhang, XiaoJun Wang

**Affiliations:** Department of Hepatobiliary Surgery, Southwest Hospital, Third Military Medical University (Army Medical University), Chongqing, China.

**Keywords:** USP10, FOXC1, Deubiquitination, Pancreatic ductal adenocarcinoma, WNT signaling

## Abstract

Increasing evidence has suggested that ubiquitin-specific protease 10 (USP10), a deubiquitinating enzyme, plays an essential role in targeted protein degradation and participates in cancer progression. However, the relationship between USP10 and pancreatic ductal adenocarcinoma (PDAC) is poorly understood. Here, we developed a USP-targeting siRNA library, combining a loss-of-function experimental screen in patient-derived PDAC cells. This approach identified USP10 as a master regulator of PDAC cell migration. High USP10 expression levels were observed in PDAC patient tissues, which were associated with poor prognosis. Furthermore, knockdown of USP10 expression inhibited PDAC cell proliferation and migration *in vivo* and *in vitro*. Mechanistically, USP10 increased FOXC1 protein stability via deubiquitination. The phosphorylation of FOXC1 at S272A was dependent on USP10-mediated deubiquitination of FOXC1. Additionally, USP10 promoted FOXC1 protein localization in the nucleus. Interestingly, FOXC1 could increase USP10 mRNA expression levels by transcriptional activation. Our data suggest that a positive feedback loop exists between USP10 and FOXC1 that can activate WNT signaling, thus facilitating PDAC malignant progression. Therefore, USP10 represents an exciting therapeutic target that could support new strategies for treating PDAC.

## Background

Pancreatic ductal adenocarcinoma (PDAC) is a pathological subtype that accounts for more than 80% of pancreatic cancer (PC) cases, which is one of the most lethal cancers worldwide 1. In contrast to the apparent progress in survival benefits for many other cancer types, the current 5-year survival rate for PC remains around 11% 2. The early systemic spread and aggressive local growth of the disease lead to this poor prognosis 3. More concerningly, a number of factors contribute to the lethality of PDAC, especially that the disease is not detected until an advanced stage, usually after distant metastasis 4. Radical resection of the tumor is essential for the systemic treatment of PDAC, but this cannot be performed on many patients because of distant metastasis 5. Therefore, exploring new sensitive metastasis-related biological targets at an early stage is crucial for increasing PDAC patient survival rates.

Protein modifications, such as ubiquitination and phosphorylation, are regulated at the post-translational level by proteases and kinases, which participate in cell biological processes including tumor malignant progression 6. Increasing recent evidence has suggested that deubiquitinating enzymes (DUBs) play an essential role in the ubiquitin-proteasome by hydrolyzing amide bonds between the single and polyubiquitin chains of substrate proteins 7. Among the DUBs encoded in the human genome, five subtypes are characterized as cysteine peptidases, of which the ubiquitin-specific protease (USP) family is the largest group 8. USP family members counteract ubiquitinase activity and affect protein functions involved in the regulation of protein stability, subcellular localization, and activity 9. For example, USP25 stabilizes and decreases hypoxia-inducible factor (HIF)-1α degradation by deubiquitination, which promotes HIF1-α transcriptional activity and facilitates HIF-1α-mediated glycolysis 10. Bhattacharya *et al.* found that silencing USP10 expression could induce endoplasmic reticulum stress-induced unfolded protein response and suppress PDAC cell viability 11. Additionally, our previous study indicated that the kinesin KIF15 could serve as scaffolding protein to recruit USP10 and PGK1, which can promote PGK1 stability by USP10 mediated deubiquitination and increase aerobic glycolysis in PDAC 12. However, the biological role and therapeutic potential of USP10 have not yet been fully explored in PDAC.

The Forkhead box C1 (FOXC1) transcription factor participates in various biological processes in both normal and tumor cells 13. The literature has indicated that abnormal FOXC1 expression patterns are associated with tumor progression and poor prognosis 14. FOXC1 positively transcriptionally regulates IGF-1R and promotes cell proliferation, migration, invasion, and angiogenesis in PC 15. Therefore, exploring the relationship between DUBs and transcription factors may help develop a new therapeutic strategy for PDAC.

In this study, we found that USP10 can interact with FOXC1 and increase its protein stability by deubiquitination. USP10 can also be transcriptionally regulated by FOXC1, leading to a positive feedback loop that is involved in regulating PDAC cell proliferation and metastasis. These data suggest that USP10 is a promising therapeutic target in PDAC.

## Materials and methods

### Human tissue samples

PC tissue samples were obtained from patients at Southwest Hospital by surgical resection with their consent and stored at -80°C. The experiments involving these samples were approved by the Ethics Committee of Southwest Hospital, Third Military Medical University (Army Medical University).

### Cell lines, reagents, and antibodies

The HPDE, PANC-1, MIA PaCa-2, SW-1990, AsPC-1, BxPC-3, and HEK-293T cell lines were obtained from the American Type Culture Collection (ATCC; Manassas, VA, USA). PANC-1, MIA PaCa-2, SW-1990, and HEK-293T cells were cultured in Dulbecco's Modified Eagle Medium (DMEM; Gibco, Grand Island, NY, USA), while HPDE, AsPC-1, and BxPC-3 cells were cultured in RPMI 1640 medium (Gibco). All media were supplemented with 10% fetal bovine serum (FBS; Invitrogen, Carlsbad, CA, USA). FH535 (a β-catenin pathway inhibitor), MG132 (a proteasome inhibitor), and chloroquine (an autophagy inhibitor) were purchased from MedChemExpress (Monmouth Junction, NJ, USA). Commercially available antibodies included the following: anti-USP10 (Proteintech, Rosemont, IL, USA; 19374-1-AP; 1:1,000), anti-FOXC1 (Thermo Fisher Scientific, Waltham, MA, USA; PA1-807; 1:1,000), anti-MYC (Proteintech; 60003-2-Ig; 1:1,000), anti-β-Tubulin (Proteintech; 10094-1-AP; 1:1,000), anti-Flag (Proteintech; 66008-4-Ig; 1:1,000), anti-GAPDH (Proteintech; 60004-1-Ig; 1:50,000), anti-MMP-9 (Proteintech; 10375-2-AP; 1:1,000), anti-MMP2 (Proteintech; 10373-2-AP; 1:1,000), ant-Snail (ABclonal, Woburn, MA, USA; A5243; 1:1,000), anti-Vimentin (Proteintech; 10366-1-AP; 1:1,000), Phospho-β-Catenin (Ser33/37/Thr41) (Cell Signaling Technology, Danvers, MA, USA; 9561; 1:1,000), and β-Catenin (Cell Signaling Technology; 8480; 1:1,000).

### RNA extraction and quantitative reverse transcription-polymerase chain reaction (qRT-PCR)

Total RNA was purified by resuspending the lysed cells in RNA Isolation Reagent (Vazyme BioTech, Nanjing, China) following the corresponding protocol. RNA concentrations were determined using a NanoDrop 2000c instrument (Thermo Fisher Scientific). Total RNA was reverse transcribed into cDNA using the HiScript cDNA synthesis kit (Vazyme BioTech). Then, qRT-PCR was performed using the SYBR green PCR mix (Vazyme BioTech). The primer sequences used in our experiments are shown in Supplementary Table 1.

### Cell transfection

A short hairpin RNA (shRNA) targeting USP10 or the negative control (NC) shRNA was constructed into the lentiviral vector pENTR/H1/TO (Thermo Fisher Scientific). Lentiviral vectors encoding the USP10 or FOXC1 gene were also constructed. The packaged viruses were then transfected into PC cells and cultured for 48 hours. Because all the lentiviruses expressed the puromycin resistance gene, the transfected cells were treated with puromycin for selection. Gene expression was validated via qRT-PCR to confirm the transfection efficiency. The corresponding shRNA sequences are included in Supplementary Table 2.

### TdT-mediated dUTP nick-end labeling (TUNEL) assay

After the cells were transfected, TUNEL assays were performed using the One Step TUNEL Apoptosis Assay Kit (Beyotime, Shanghai, China) following the manufacturer's protocols. Images of stained cells were captured by fluorescence microscopy, with quantification performed using Image J software (NIH, Bethesda, MD, USA).

### Colony formation assay

Cells from different groups were seeded in 6-well plates (500 cells/well) with at least three replicate wells and cultured with complete medium at 37°C for 2 to 3 weeks. For visualization, 4% paraformaldehyde (PFA) was used to fix the cultured cells for 15 minutes at room temperature, followed by staining with 2% crystal violet for 20 minutes. Images were photographed and the number of colonies was calculated.

### Cell migration and invasion assays

For wound healing assays to assess cell migration, PC cells were seeded in 6-well plates and cultured until they were 80% to 90% confluent. The cells were then scratched with a 200 μL pipette tip in the middle of each well, after which they were cultured with serum-free medium for 24 hours to monitor the cell migration patterns. Five distinct wound site fields were randomly chosen, with cell migration measured using a microscope and Image J software. The percentage of wound gap closure was calculated as the ratio of the residual wound area to the original wound area.

For Transwell migration assays, 5×10^4^ PC cells were seeded in the upper chamber of the plates (BD BioCoat; BD Biosciences, Franklin Lakes, NJ, USA) in 200 μL serum-free medium, while 10% FBS was added to the lower chamber. After culturing for 36 hours, the cells that migrated from the upper chamber to the lower chamber were fixed with 4% PFA and stained with 0.1% crystal violet, then counted using a microscope.

For Transwell invasion assays, the upper chamber was coated with matrigel (BD Biosciences). Then, 5×10^4^ PC cells were seeded in the upper chamber in 200 μL serum-free medium, while the lower chamber contained 700 μL medium with 10% FBS. The subsequent steps were the same as the Transwell migration assay. For both the migration and invasion assays, five random cell fields were counted and the experiments were performed in triplicate.

### Immunofluorescence (IF) assay

For IF analysis, different groups of PC cells were seeded into multi-chamber slides and incubated at 37°C. After the cells were 80% confluent, the slides were fixed in 4% PFA for 20 minutes at room temperature and permeabilized with 0.1% Triton X-100. The samples were then incubated with primary antibodies, followed by incubation with secondary antibodies. After the cell samples were fixed and stained with DAPI, images were captured by fluorescence microscopy.

### Western blot analysis

Total protein was extracted from cell lysates using RIPA buffer with protease inhibitor cocktail and phosphatase inhibitors. Then, the protein samples were heated in SDS loading buffer for 5 to 10 minutes at 98°C. The protein levels of each group were analyzed by standard western blot analysis procedures. The GAPDH or tubulin protein levels were used as an internal reference to normalize gene expression in corresponding experiments.

### Co-immunoprecipitation (Co-IP)

For Co-IP experiments, protein samples were extracted from PC cells using NP40 lysis buffer. The protein A/G magnetic beads (MedChemExpress) were first washed in the IP buffer at least four times in preparation for the following steps. The protein concentration of 10% of the lysate was determined using a BCA kit (Biosharp, Shanghai, China). The lysates were then incubated overnight at 4°C with the corresponding IP antibody or IgG isotype control followed by incubation with the magnetic beads. The immunoprecipitates were analyzed using western blots, as described above.

### Ubiquitination assay

For *in vivo* ubiquitination assays, the indicated plasmids were transfected into 293T cells for 24 hours, then the efficiency was verified using western blot analysis. Next, 20 μM MG132 was added into the medium to inhibit proteasome activity. The cells were then lysed using NP40 buffer and immunoprecipitated with an anti-Flag or anti-FOCX1 antibody. The immunoprecipitates were then subjected to western blot analysis.

### Mass spectrometry analyses

Liquid chromatography tandem mass spectrometry (LC-MS/MS) was used for mass spectrometry analyses in this study. PC cells were treated with NP40 buffer to extract the total protein. The lysates were then incubated with an anti-USP10 antibody or control IgG for IP. The immunoprecipitated proteins were then digested with modified sequencing grade trypsin, which purified the truncation of proteins for analysis. The fragmented peptides were then analyzed by LC-MS/MS to identify USP10-interacting proteins.

### Chromatin immunoprecipitation (ChIP)

For ChIP assays, the cells were lysed using ChIP lysis buffer and the crosslinked chromatin and protein complexes were then lysed by sonication. Appropriate amounts of samples were stored as input DNA samples. An anti-FOXC1 antibody or normal rabbit IgG was used for IP. Then, qPCR was used to analyze FOXC1 binding to the USP10 promoter, with the results normalized to the input samples. The primer sequences used for qPCR are shown in Supplementary Table 3.

### Immunohistochemistry (IHC)

The expression levels of the indicated proteins were examined in tumor and normal samples using IHC analysis of a tissue microarray. The samples were incubated with anti-USP10, anti-Ki-67, anti-PCNA, and anti-FOXC1 antibodies, then incubated with a horseradish peroxidase-conjugated secondary antibody. The samples were visualized with DAB and counterstained with hematoxylin, then the images were captured using a light microscope.

### Dual-luciferase reporter assay

To assess the regulatory effects of FOXC1 on USP10 mRNA, dual-luciferase reporter assays were performed as described previously 16. The USP10 promoter region from 2000 bp upstream to 1 bp downstream of the transcription start site was cloned into the pGL3-Basic firefly luciferase reporter plasmid (Promega, Madison, WI, USA). The relative luciferase activity was calculated by normalizing the firefly luciferase activity to the Renilla luciferase activity.

### Animal experiments

For the subcutaneous xenograft experiments, six-week-old female BALB/c-nude mice (specific-pathogen-free) were purchased from Vital River (Beijing Vital River Laboratory Animal Technology Co., Ltd., Beijing, China). The mice were randomly divided into three groups and subcutaneously inoculated with sh-control, sh-USP10-1, or sh-USP10-2 PANC-1 cells (5×10^6^ cells/100 µL) to establish the xenograft PDAC tumor-bearing model. Tumor sizes and volumes were monitored every three days. The tumor volume was calculated using the following formula: V (cm^3^) = 1/2 × length × width^2^.

For the metastasis model, six-week-old female BALB/c-nude mice were randomly divided into three groups. Each mouse underwent surgery without pain under anesthesia. The abdominal cavity was opened successively to expose the spleen, then the cell suspension (5×10^6^ cells/100 µL) was slowly injected into the spleen. The abdomen was then closed layer by layer and the mouse was woken up from anesthesia. After ten weeks of feeding, the mice were painlessly sacrificed. The liver and lung tissues were dissected and measured by section observation under a microscope. All the tumor, liver, and lung tissues were paraffin-embedded. Tissue sections were analyzed using IHC assays and hematoxylin and eosin (H&E) staining, with the results observed by microscopy.

All animal experiments were approved by the Southwest Hospital, Third Military Medical University (Army Medical University) Animal Care and Use Committee.

### Bioinformatics analysis

The datasets used in this study were obtained from The Cancer Genome Atlas (TCGA; https://portal.gdc.cancer.gov) and Gene Expression Omnibus (GEO; https://www.ncbi.nlm.nih.gov/geo/) databases, including TCGA-PAAD, GSE62452, GSE16515, and GSE130221. All data were analyzed using R software (version 4.0.1).

### Statistical analysis

SPSS 22.0 software (Chicago, IL, USA) and GraphPad Prism 9.0 (La Jolla, CA, USA) were used to analyze the data. The t-test or one-way ANOVA was used to analyze the significant differences between groups. The Cox proportional risk regression model was used to assess the prognostic variables of overall survival (OS) and recurrence-free survival. The data are presented as the mean ± standard deviation (SD) from three individual experiments. A *P*-value < 0.05 was considered statistically significant.

## Results

### USP10 is highly expressed in PC and positively correlated with poor prognosis

**Figure 1:**
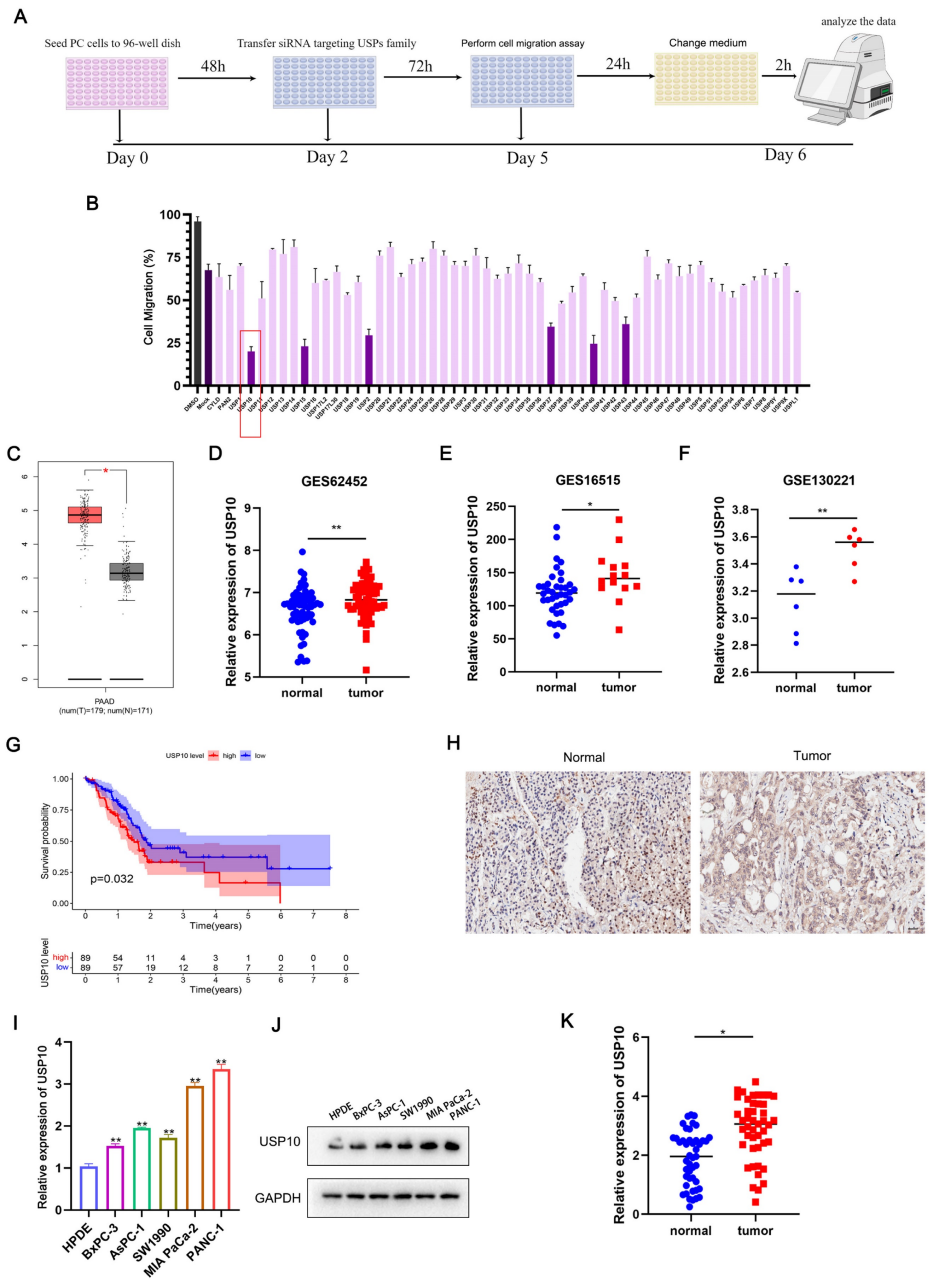
** Screening and identification of USPs in pancreatic cancer.** (a) The siRNA library of USPs were constructed and combined with migration assays to screen and identify the potential USPs which were associated with cell migration in PDAC. (b) Six USPs (USP10, USP15, USP2, USP37, USP40, USP43) were identified the potential USPs. (c) TCGA-PAAD dataset were performed to identify the expression of USP10 in PDAC tissues and normal tissues. (d-f) GEO dataset (GES62452, GES16515, GSE130221) were performed to further identify the expression of USP10 in PDAC tissues and normal tissues. (g) Individuals in the TCGA-PAAD dataset were divided into the USP10 high expression and USP10 low expression groups according to the expression of USP10. The overall survival rate of the PDAC patients was estimated by Kaplan-Meier analysis. (h) Tissues chips were performed to detect the expression of USP10 in PDAC tissues (scale label:100μm). (i-j) The expression of USP10 in HPDE and five pancreatic cancer cell lines, including AsPC-1, BxPC-3, MIA PaCa-2, PANC-1 and SW1990 were validated by qRT-PCR analysis and western blot. (k) The expression of USP10 in our collected PDAC tissues from patients were evaluated by RT-qPCR analysis. *, and ** respectively represent *p*<0.05 and *p*<0.01.

To identify the key USP involved in regulating PDAC cell migration, we conducted a functional experiment using a USP-specific siRNA library. After transfecting the siRNAs targeting the USP family, the expression levels of USPs were successfully knocked down. We then performed an unbiased loss-of-function screening with Transwell assays. Here, the individual knockdown of six DUBs resulted in a more than two-fold decrease in the cell migration rate. Among them, USP10 knockdown showed the strongest inhibitory effect on PDAC cell migration (Figure 1A/B).

Further analysis of TCGA data indicated that USP10 was significantly overexpressed in PDAC tissues compared with paired normal pancreatic tissues (Figure 1C). USP10 also showed higher mRNA expression levels in PDAC tissues compared with normal pancreatic tissues in GEO datasets (GES62452, GES16515, GSE130221) (Figure 1D-F). Subsequently, Kaplan-Meier survival analysis showed that the TCGA-PAAD patients with higher USP10 mRNA expression levels had shorter OS rates and an unfavorable prognosis (Figure 1G). To further confirm USP10 expression patterns in PDAC, a tissue microarray was examined, which suggested that the relative USP10 expression levels were higher in PDAC tissues (Figure 1H). In parallel experiments, qRT-PCR and western blot analyses were performed to respectively verify the USP10 mRNA and protein expression patterns in PDAC cell lines (BxPC-3, AsPC-1, SW1990, MIA PaCa-2, PANC-1) and a human pancreatic ductal cell line (HPDE). The results indicated that USP10 showed higher mRNA and protein expression levels in PDAC cell lines compared with HPDE cells (Figure 1I/J). The subsequent analysis of human PDAC tissues collected in our hospital revealed that USP10 expression was much higher in the PDAC specimens than in the paired normal pancreatic tissues (Figure 1K). These data demonstrated that USP10 is a potential oncogene and overexpressed in PDAC. We also expanded the research samples to explore the clinical relevance, finding that high USP10 expression levels are often associated with lymph node metastasis (Table 1). However, there was no significant correlation between USP10 expression and tumor size, differentiation degree, or TNM stage (Table 1).

### USP10 promotes PC cell proliferation *in vitro* and *in vivo*

**Figure 2:**
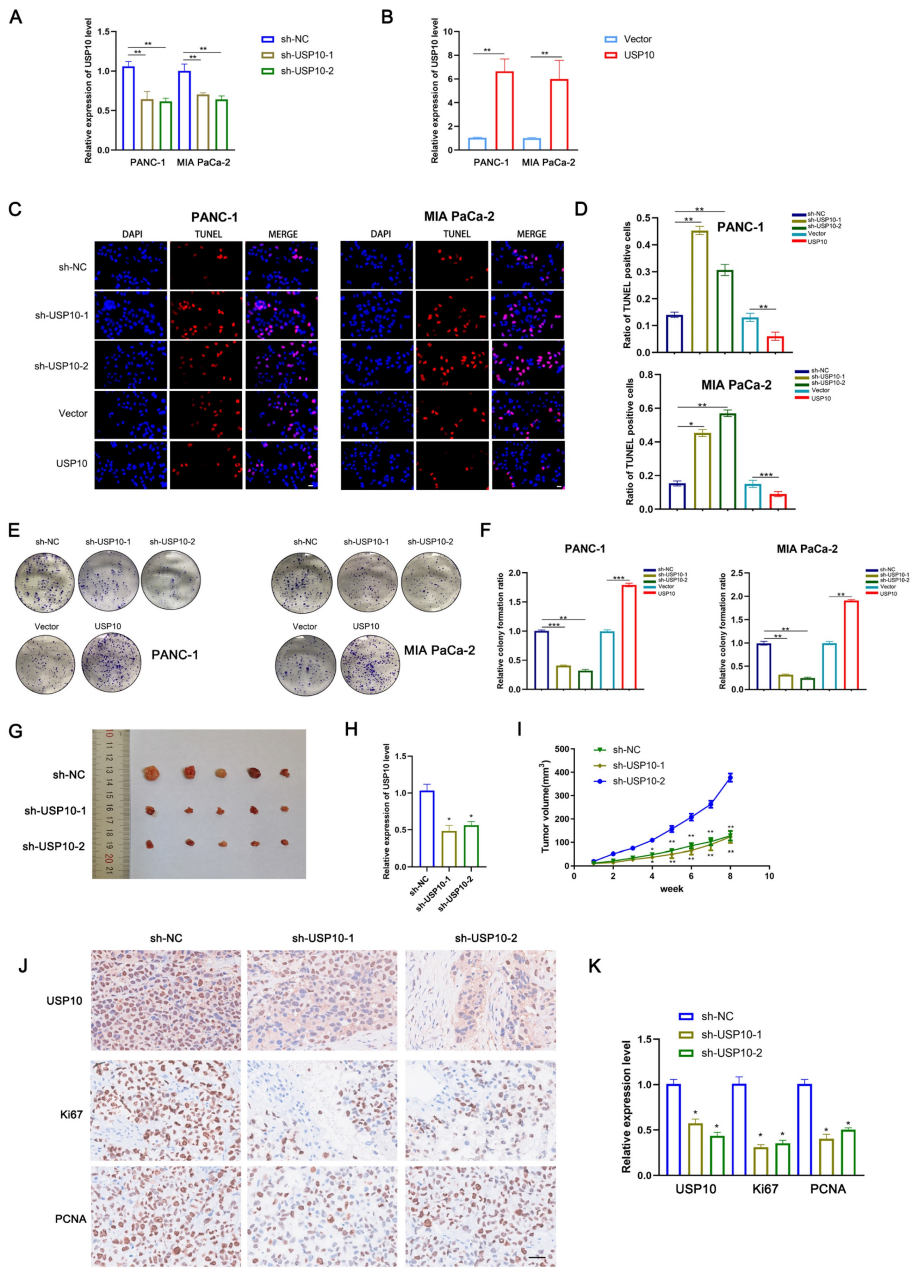
** USP10 promotes pancreatic cancer proliferation *in vitro* and *in vivo*.** (a,b) The infection efficiency of USP10 overexpression and knockdown lentivirus was validated by qRT-PCR analysis. (c,d) The effect of USP10 on cell apoptosis in PDAC cells were measured by Tunel assays. (e,f) The effect of USP10 on cell proliferation in PDAC cells were measured by colony forming assays. (g) The 6-weeks old female BALB/c-nude mice were randomly divided into three groups (n=5) and subcutaneously inoculated with sh-control, sh-USP10-1 or sh-USP10-2 PANC-1 cells (5×10^6^ cells/100 µl) to established the xenograft PDAC bearing model. The photograph of tumors sh-NC, sh-USP10-1 and sh-USP10-2 groups. (h) The relative expression of USP10 in each groups were detected by PCR analysis. (i) The tumor volume curve in each groups. (j,k) The immunohistochemical method was performed to confirmed the expression of USP10, Ki67 and PCNA in each groups. *, ** and *** respectively represent *p*<0.05, *p*<0.01 and *p*<0.001.

Because our data suggested that USP10 might be an oncogene in PDAC, we next assessed its biological functions in this disease. First, we successfully generated stable overexpression and knockdown cell lines by respectively using lentiviral vectors encoding USP10 or a USP10-targeting shRNA (Figure 2A/B). TUNEL assays were performed to examine the levels of apoptosis in PDAC cells following USP10 overexpression or knockdown. The results suggested that USP10 overexpression significantly suppressed the levels of apoptosis, while USP10 knockdown had the opposite effect (Figure 2C/D). Moreover, colony formation assays were performed to evaluate the effect of USP10 expression on cell proliferation. Interestingly, USP10 overexpression significantly promoted both cell proliferation and colony formation. However, USP10 knockdown resulted in a decreased number of colonies and inhibited the cell proliferation rate (Figure 2E/F). Similar effects were observed in animal experiments, which showed smaller subcutaneous tumor sizes in the USP10 knockdown groups than in the USP10 negative control group (Figure 2G). Subsequently, qRT-PCR analysis was performed to detect USP10 mRNA expression levels in subcutaneous tumors from the different groups. The USP10 knockdown groups displayed lower USP10 expression levels (Figure 2H). Notably, the tumor volume curve indicated that USP10 knockdown significantly inhibited PDAC growth (Figure 2I). IHC assays were performed to confirm the protein expression patterns of Ki67 and PCNA, which are two markers that can reflect the strength of cell proliferation. The results suggested that Ki67 and PCNA both showed higher expression levels in the negative control group compared with the USP10 knockdown groups. Collectively, these data provided evidence that USP10 can promote PDAC cell proliferation (Figure 2J/K).

### USP10 promotes PC cell migration and invasion *in vitro* and *in vivo*

**Figure 3:**
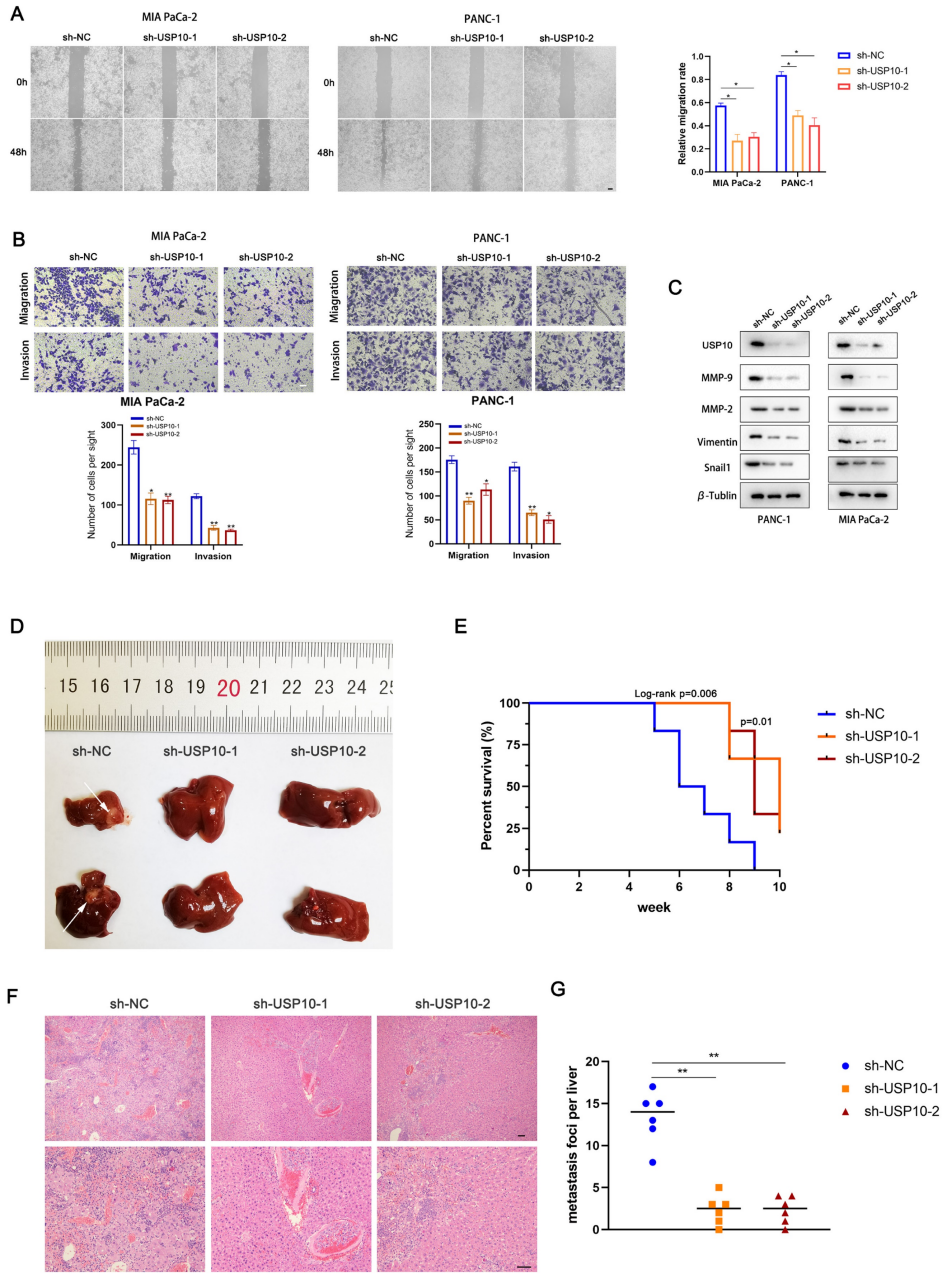
**USP10 promotes pancreatic cancer migration and invasion *in vitro* and *in vivo.*** (a) Wound healing assay was performed to measure the migrated abilities of PDAC cells transfected with sh-USP10. (b) Transwell assays were performed to detect the migration and invasion of PDAC cells transfected with sh-USP10. (c) Western blot analysis was performed to detect the expression of MMP-2, MMP-9, Vimentin and Snail1. (d) The 6-weeks old female BALB/c-nude mice were randomly divided into three groups (n=6) which were respectively injected with lentivirus contain sh-NC, sh-USP10-1 and sh-USP10-2 sequences to establish metastasis model. Representative mice livers were taken photos. (e) The overall survival of mice were evaluated by Kaplan-Meier analysis. (f,g) The livers of mice were dissected and measured by section observation under microscopy.

We next aimed to assess if USP10 plays an essential role in PDAC cell migration and invasion. Wound healing assays were performed to examine the migration abilities of PDAC cells following USP10 expression manipulation. The results revealed that USP10 knockdown in PANC-1 and MIA PaCa-2 cells significantly inhibited their migration (Figure 3A). Moreover, data from the Transwell assays corroborated the wound healing assay results. These findings also suggested that USP10 knockdown decreased the PDAC cell migration and invasion rates (Figure 3B). Furthermore, western blot analysis was performed to evaluate the effect of USP10 expression on the protein expression levels of MMP2, MMP9, Vimentin, and Snail1. Of note, the expression patterns of these abovementioned proteins were consistent with USP10 expression (Figure 3C). USP10 knockdown significantly decreased the expression levels of these proteins, suggesting that USP10 can potentially promote the epithelial-mesenchymal transition (EMT) and extracellular matrix degradation, further supporting that USP10 can promote PDAC migration and invasion. Next, we developed metastasis models in nude mice using stable low expression of USP10 and negative controls. The results revealed that USP10 knockdown successfully decreased the number of liver metastases (Figure 3D). Moreover, Kaplan-Meier survival analysis demonstrated that the negative control group mice showed a shorter OS than those in the USP10 knockdown groups (Figure 3E). H&E staining showed that USP10 knockdown resulted in fewer metastases from the microcosmic perspective (Figure 3F/G). These results illustrated that USP10 can promote PDAC migration and invasion *in vitro* and *in vivo*.

### USP10 interacts with FOXC1 and positively regulates FOXC1 protein levels

**Figure 4:**
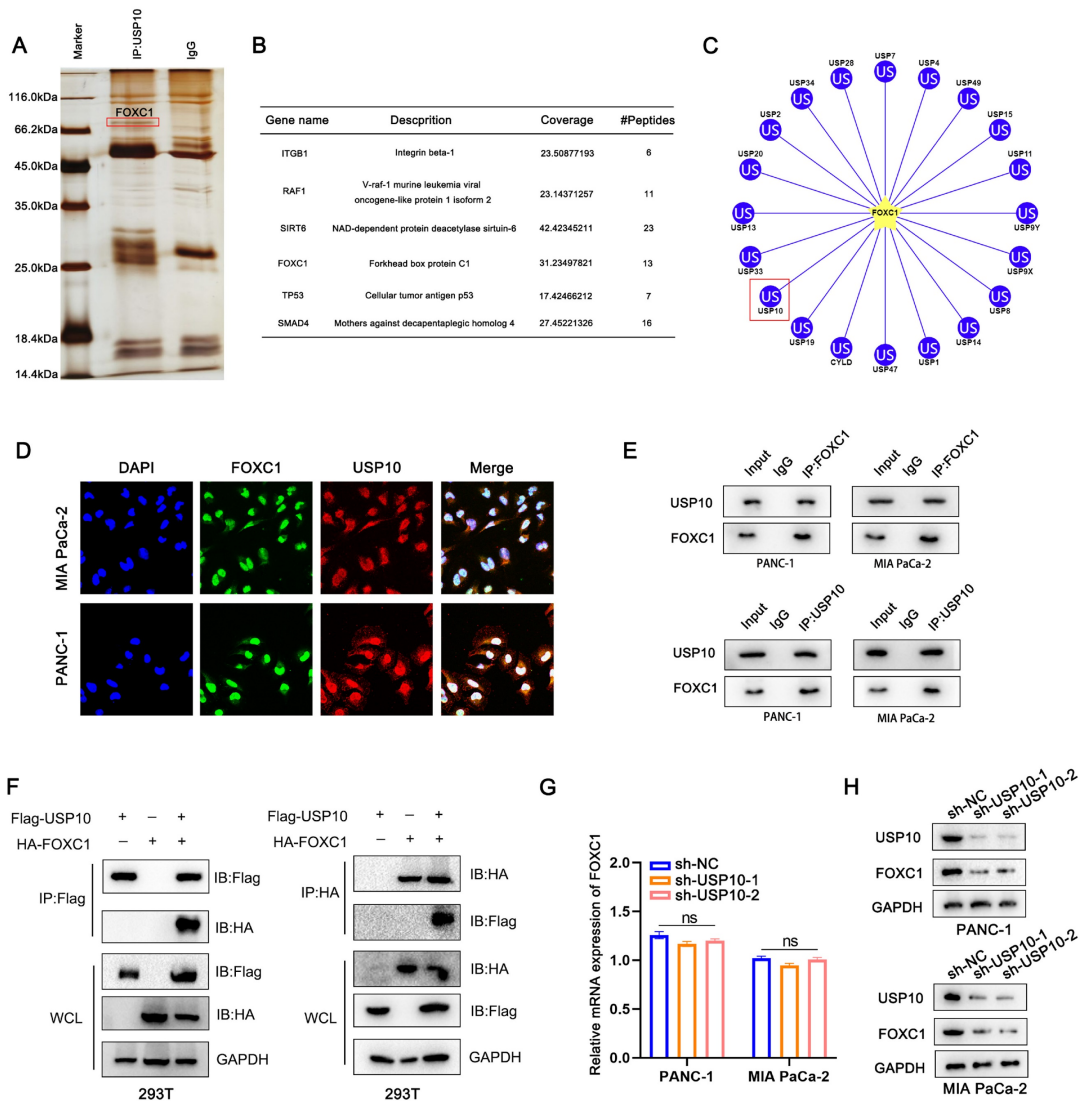
** USP10 interacts with FOXC1 and positively change the protein level of FOXC1.** (a) The Co-IP was performed to pull down the interacting proteins of USP10 then analyzed by LC-MS, the silver stain shows potential proteins that can bind. (b) The following proteins rank high on the list: ITGB1, RAF1, SIRT6, FOXC1, TP53 and SMAD4. (c) Ubibrower database (http://ubibrowser.bio-it.cn/ubibrowser_v3/) were performed to predict the potential binding DUPs of FOXC1. (d) Immunofluorescence was performed to clarify the co-location of USP10 and FOXC1. (e) Co-IP analysis was performed to test the interaction between endogenous USP10 and FOXC1. (f) Co-IP analysis was further performed to test the interaction between exogenous USP10 and FOXC1. (g) PCR analysis were performed to detect the mRNA expression of FOXC1 after USP10 knockdown. (h) Western blots were performed to detect the protein expression of FOXC1 after USP10 knockdown.

The multiple biological functions of DUBs are mainly dependent on their interacting target proteins 17. To investigate the potential target proteins, we used a lentivirus encoding USP10 to increase USP10 expression levels in cells, then performed Co-IP experiments to pull down the proteins interacting with USP10 and analyzed them by LC-MS. The following proteins ranked highest: ITGB1, RAF1, SIRT6, FOXC1, TP53, and SMAD4 (Figure 4A/B). Bioinformatics analysis using the ubibrower database (http://ubibrowser.bio-it.cn/ubibrowser_v3/) allowed us to narrow down the putative substrate proteins of USP10. We excluded the proteins previously known to bind to USP10. We also found FOXC1 to be a potential USP10 substrate protein by LC-MS (Figure 4C), so we subsequently focused our experiments on this most promising candidate. IF assays were performed to further confirm the interaction between USP10 and FOXC1 and examine their localization. The results illustrated that USP10 co-localized with FOXC1, mainly accumulating in the nucleus (Figure 4D). Next, we used endogenous and exogenous Co-IP experiments to detect if these proteins directly interact with each other. Notably, the results confirmed that FOXC1 could interact with USP10 via endogenous and exogenous Co-IPs (Figure 4E/F). To further assess if USP10 could affect FOXC1 expression patterns, PCR and western blot analyses were performed. Interestingly, the results indicated that knocking down USP10 expression had a more significant effect on FOXC1 protein levels compared with its mRNA levels (Figure 4G/H), with USP10 knockdown leading to decreased FOXC1 protein expression (Figure 4H). Additionally, USP10 overexpression significantly increased the protein levels, but not the mRNA levels, of FOXC1 (Supplementary Figure 1A/B). Furthermore, we found that FOXC1 was overexpressed in TCGA-PAAD tissues compared with normal tissues, with these higher FOXC1 expression levels being associated with shorter patient OS and disease-free survival rates (Supplementary Figure 2A-C). PCR and western blot analyses both confirmed that FOXC1 was highly expressed in PDAC cell lines and tissues (Supplementary Figure 2D-F). Next, we successfully transfected a FOXC1-targeting shRNA into PANC-1 and MIA PaCa-2 cell lines (Supplementary Figure 2G). Colony formation and TUNEL assays suggested that FOXC1 knockdown led to inhibited proliferation and increased apoptosis in PDAC cells (Supplementary Figure 2H/I). Overall, this evidence suggested that USP10 can potentially exert carcinogenic effects by interacting with and stabilizing FOXC1 protein.

### USP10 deubiquitinates and stabilizes FOXC1 by attenuating its degradation

Our data suggested that decreased USP10 expression levels led to reduced protein expression, but not mRNA expression, of FOXC1. To investigate how USP10 can affect FOXC1 protein expression patterns, we examined the effects of USP10 on FOXC1 in the autophagy and proteasome pathways. Chloroquine is an inhibitor that can induce cell autophagy in PDAC 18.

**Figure 5:**
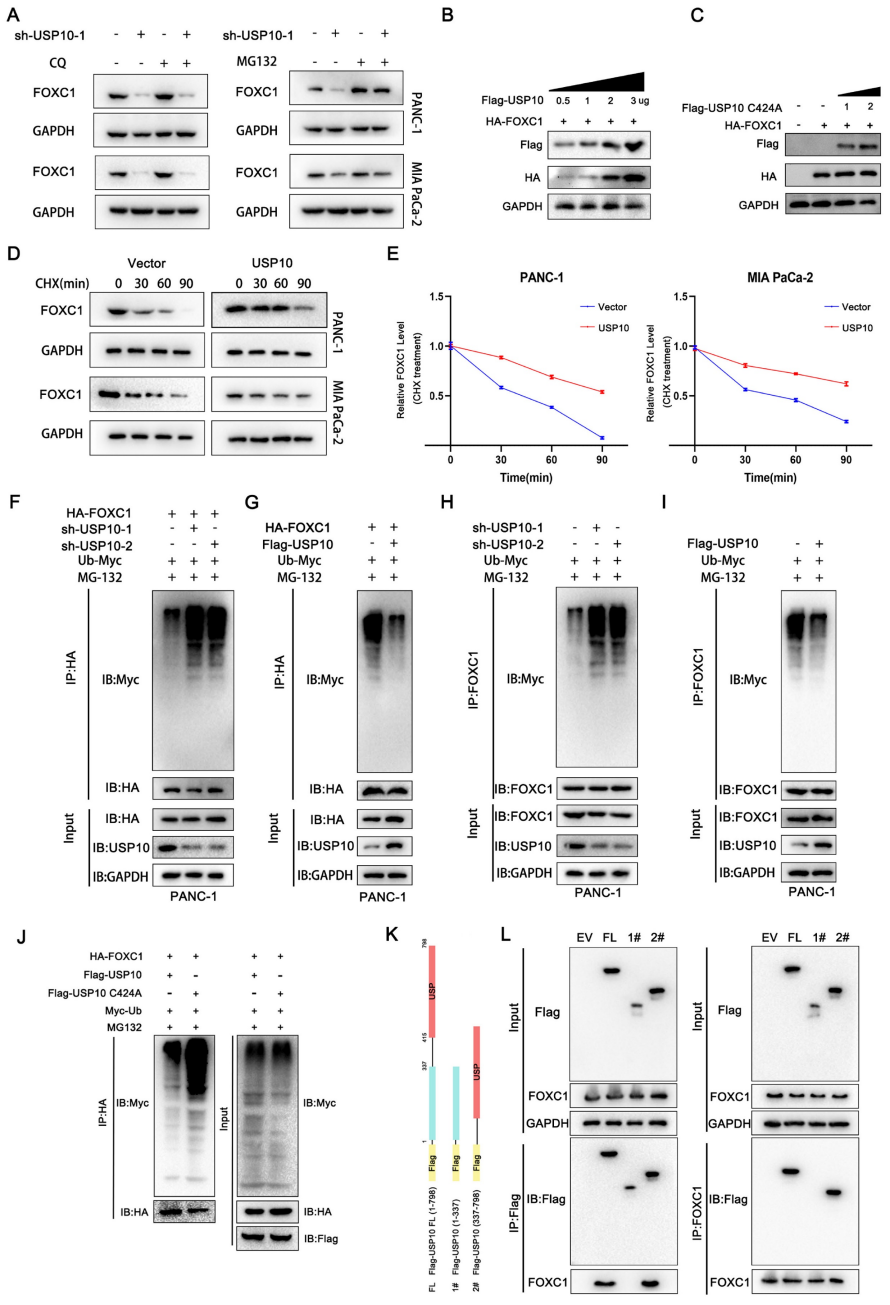
**USP10 deubiquitinates and stabilizes FOXC1 by attenuating the degradation of FOXC1.** (a) Chloroquine (CQ 10 μM) and proteasome inhibitor MG132 (10 μM) were performed to measure whether USP10 knockdown change the expression of FOXC1. (b) Effects of different concentrations of USP10 on FOXC1 protein expression. (c) Effects of USP10 C242A on FOXC1 protein expression. (d) Protein stability assay by using cycloheximide (CHX, 50 μg/mL) to treat cell for different time was performed to evaluate the effect of USP10 overexpression, followed by western blot analysis. (e) The protein stability curve of FOXC1 after CHX treatment. (f-i) The ubiquitination of FOXC1 in USP10 knockdown or USP10 overexpression was analyzed by Co-IP with treatment of MG132 and Myc-Ub. (j) The ubiquitination of FOXC1 in USP10 wild type or USP10 C424A mutant type was analyzed by Co-IP with treatment of MG132 and Myc-Ub. (k,l) The truncated of USP10 and FOXC1 was co-transfected into 293T cells, followed by Co-IP assay to clarify the interaction domain between FOXC1 and USP10.

We therefore aimed to use chloroquine to reverse the reduced FOXC1 expression levels in the sh-USP10 group. However, chloroquine treatment had little effect on FOXC1 expression in this group (Figure 5A). In addition, we found that USP10 knockdown diminished FOXC1 expression, which could be blocked by using MG132, a proteasome inhibitor (Figure 5A). These results suggested that USP10 potentially affects FOXC1 protein expression through the ubiquitination pathway, which may also be related to the deubiquitination function of USP10. Subsequently, we found that increased USP10 expression could promote higher FOXC1 expression levels following transfection of wild-type USP10 (Figure 5B). However, after transfecting mutant USP10 (USP10 C424A), which lost its deubiquitinating enzyme activity, no effects on FOXC1 expression were observed (Figure 5C). We then examined if USP10 is involved in regulating FOXC1 protein stability. PANC1 and MIA PaCa-2 cells infected with a USP10-encoding or empty lentivirus were treated with the protein synthesis inhibitor cycloheximide (CHX, 40 μM) for the indicated times, then the FOXC1 protein expression levels were analyzed. The results revealed that USP10 overexpression markedly enhanced the protein stability of FOXC1 (Figure 5D/E). From these results, we speculated that USP10 can promote FOXC1 protein stability to increase its expression levels via the ubiquitination pathway. To test this hypothesis, the ubiquitination levels were detected after infection with a lentivirus encoding USP10 or shUSP10 from both endogenous and exogenous perspectives. The exogenous experimental results indicated that after co-transfection of HA tagged-FOXC1 with sh-USP10 or Flag tagged-USP10, the USP10 knockdown groups displayed significantly increased FOXC1 ubiquitination, while the USP10 overexpression groups showed more FOXC1 deubiquitination (Figure 5F/G). Consistently, the endogenous results also suggested that USP10 knockdown facilitated FOXC1 ubiquitination, while USP10 overexpression increased FOXC1 deubiquitination (Figure 5H/I). We subsequently detected the FOXC1 ubiquitination levels following co-transfection of wild-type USP10 (Flag-USP10) or mutant USP10 (Flag-USP10 C424A) with HA-FOXC1 in HEK 293T cells. Notably, with MG132 treatment, USP10 C424A promoted FOXC1 ubiquitination compared with wild-type USP10 (Figure 5J). To determine the key domain for the interaction between FOXC1 and USP10, we constructed full-length USP10 and truncated bodies with or without the USP domain (Figure 5K). The Co-IP results indicated that FOXC1 could interact with the full-length USP10 and USP10 (337-798), which contained the USP domain (Figure 5L).

### USP10 promotes FOXC1 phosphorylation

**Figure 6:**
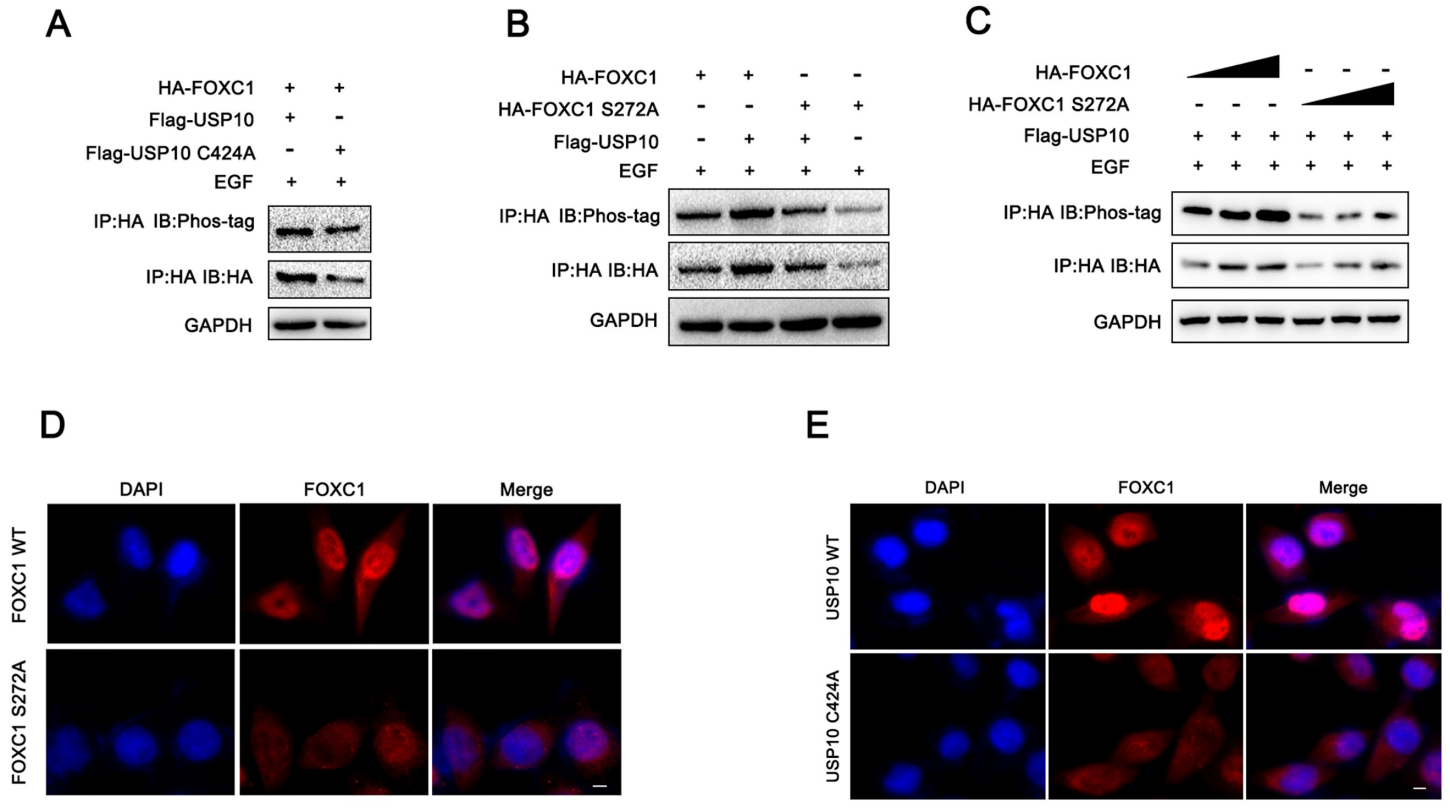
** USP10 promotes the phosphorylation of FOXC1.** (a) PDAC cells were treated with epidermal growth factor (EGF, 100 ng/ml) treatment for 5 min, then western blot analysis was performed to evaluate the effect of USP10 and USP10 C424A on the phosphorylation levels of FOXC1. (b) PDAC cells were treated with epidermal growth factor (EGF, 100 ng/ml) treatment for 5 min, then western blot analysis was performed to evaluate the effect of FOXC1 wild type and FOXC1 S272A mutant type on the phosphorylation levels of FOXC1. (c) PDAC cells were treated with epidermal growth factor (EGF, 100 ng/ml) treatment for 5 min, then western blot analysis was performed to evaluate the effect of the observed phosphorylation differences in the WT and MUT groups. (d) the effects of FOXC1 and FOXC1 S272A on the location of FOXC1. (f) the effects of USP10 and USP10 C424A on the location of FOXC1.

Previous studies have reported that serine 272 is a critical residue for maintaining proper FOXC1 protein stability 19, 20. In addition, our current data demonstrated that USP10 could inhibit FOXC1 protein degradation and promote its stability by deubiquitination. From this, we aimed to determine if USP10 can affect FOXC1 phosphorylation levels. Several reports have shown that ERK1/2 and P38 are the main kinases that phosphorylate FOXC1 19-21. Here, we treated cells with epidermal growth factor (EGF, 100 ng/mL) for 5 minutes, then examined the effect of USP10 on FOXC1 phosphorylation levels. The results suggested that overexpressing wild-type USP10 led to significantly decreased FOXC1 phosphorylation levels compared with overexpressing the USP10 C424A mutant (Figure 6A). In addition, cells transfected with mutant FOXC1 (S272A) resulted in downregulated FOXC1 phosphorylation levels, with USP10 overexpression partially rescuing this effect (Figure 6B). To ensure the reliability of the observed phosphorylation differences, we redesigned the experiment and found that the phosphorylation levels increased with HA-FOXC1 expression in the wild-type group, but not in the mutant HA-FOXC1 (S272A) group (Figure 6C). Additionally, we found that FOXC1 S272A promoted FOXC1 protein retention in the nucleus, while wild-type FOXC1 partially caused translocation to the cytoplasm (Figure 6D). Interestingly, we also observed that USP10 C424A could increase FOXC1 nuclear translocation compared with wild-type USP10 (Figure 6F).

### FOXC1 promotes USP10 expression and activates the WNT signaling pathway

**Figure 7:**
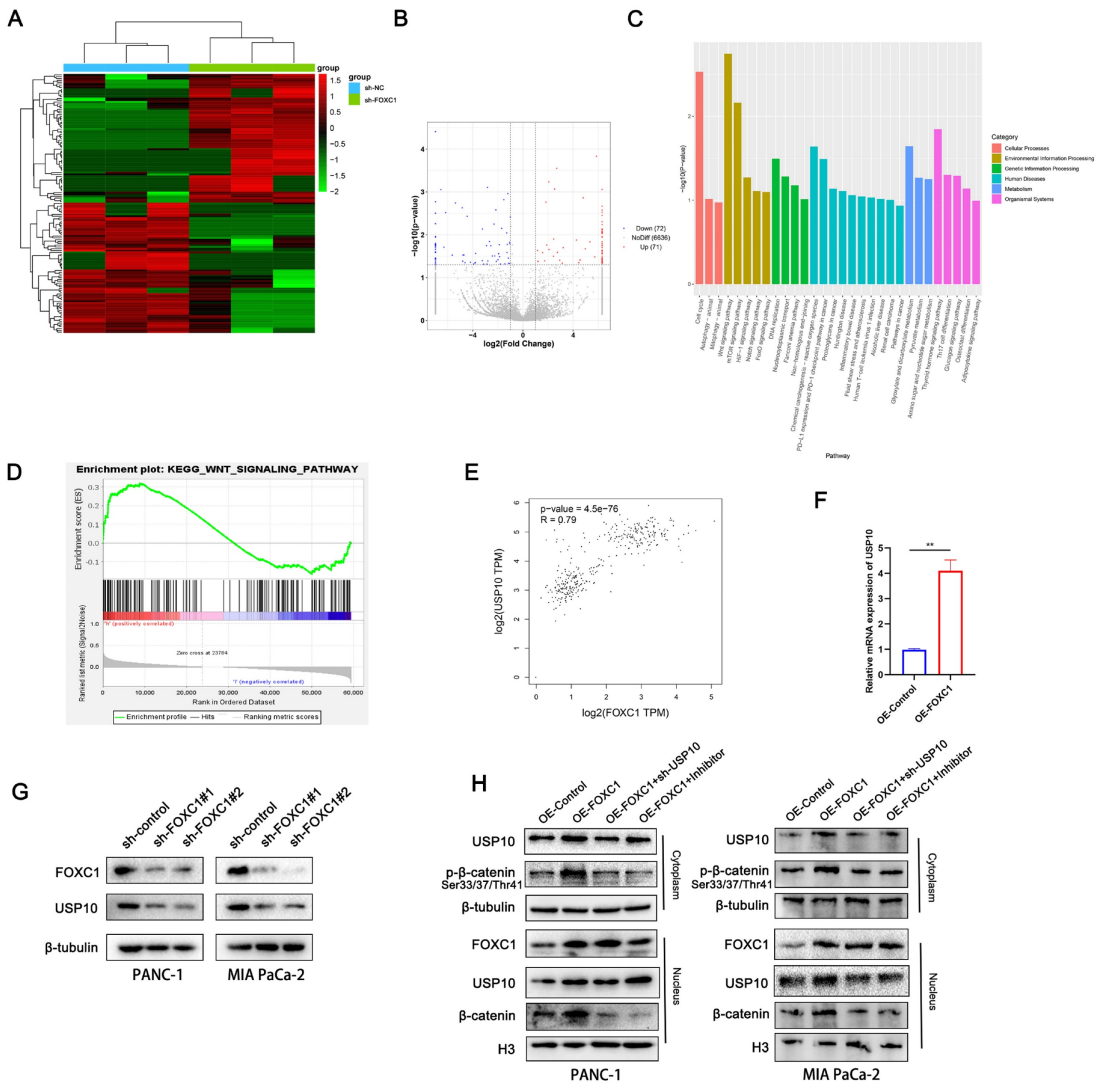
** FOXC1 promotes the expression of USP10 and activates WNT signaling pathway.** (a) RNA-seq using three paired sh-NC and sh-FOXC1 cells were performed to measure the differential genes. (b) volcano plot showed 72 genes was significantly knockdown, however, 71 genes were significantly upregulated. (c) The differentially expressed genes (DEGs) were identified and analyzed via Kyoto Encyclopedia of Genes and Genomes (KEGG) and Gene Ontology (GO) analyses. (d) Gene set enrichment analysis (GSEA) of TCGA datasets were performed to confirm the relationship between WNT signaling and FOXC1. (e) The correlation analysis between FOXC1 and USP10 was based on TCGA datasets. (f) the effects of FOXC1 on the mRNA levels of USP10 were measured by PCR analysis. (g) the effects of FOXC1 on the protein levels of USP10 were measured by WB analysis. (h) the effects of FOXC1 and USP10 on the activation of WNT signaling were measured by WB analysis. The WNT signaling inhibitor FH535 (20 μM) was used to inhibit the WNT signaling.

To investigate the mechanism by which USP10 promotes PDAC progression, we analyzed the signaling pathways that the downstream FOXC1 protein is potentially involved in. Using RNA sequencing (RNA-seq) analysis with three paired sh-NC and sh-FOXC1 cells, differentially expressed genes were identified and analyzed via Kyoto Encyclopedia of Genes and Genomes (KEGG) and Gene Ontology (GO) analyses. We found that the WNT, mTOR, HIF-1, Notch, and FoxO signaling pathways were associated with FOXC1 (Figure 7A-C). Additionally, gene set enrichment analysis (GSEA) of TCGA datasets showed that the WNT pathway was closely correlated with FOXC1 expression patterns in PDAC (Figure 7D). Therefore, we determined the WNT pathway to be a possible candidate signaling pathway for FOXC1 activation. We also found that FOXC1 downregulation resulted in decreased USP10 mRNA expression levels by RNA-seq (Figure 7B), which was consistent with TCGA data showing that FOXC1 mRNA expression levels were positively associated with those of USP10 (Figure 7E). PCR analysis then revealed that upregulating FOXC1 remarkably elevated USP10 mRNA expression levels in PANC-1 cells (Figure 7F). Next, western blot analysis suggested that knocking down FOXC1 expression led to lower protein expression levels of both FOXC1 and USP10 (Figure 7G). Western blot analysis also demonstrated that FOXC1 upregulated phosphorylated β-catenin in the cytoplasm and β-catenin in nucleus, as well as increased USP10 expression in both the cytoplasm and nucleus, suggesting that FOXC1 activated the WNT signaling pathway (Figure 7H). However, sh-USP10 and the WNT signaling inhibitor FH535 could partially reverse the activity of FOXC1 and weaken WNT signaling pathway activation (Figure 7H). These results suggested that USP10 could activate WNT signaling by FOXC1, with FOXC1 and USP10 potentially being part of a positive feedback loop.

### FOXC1 can transcriptionally activate USP10

**Figure 8:**
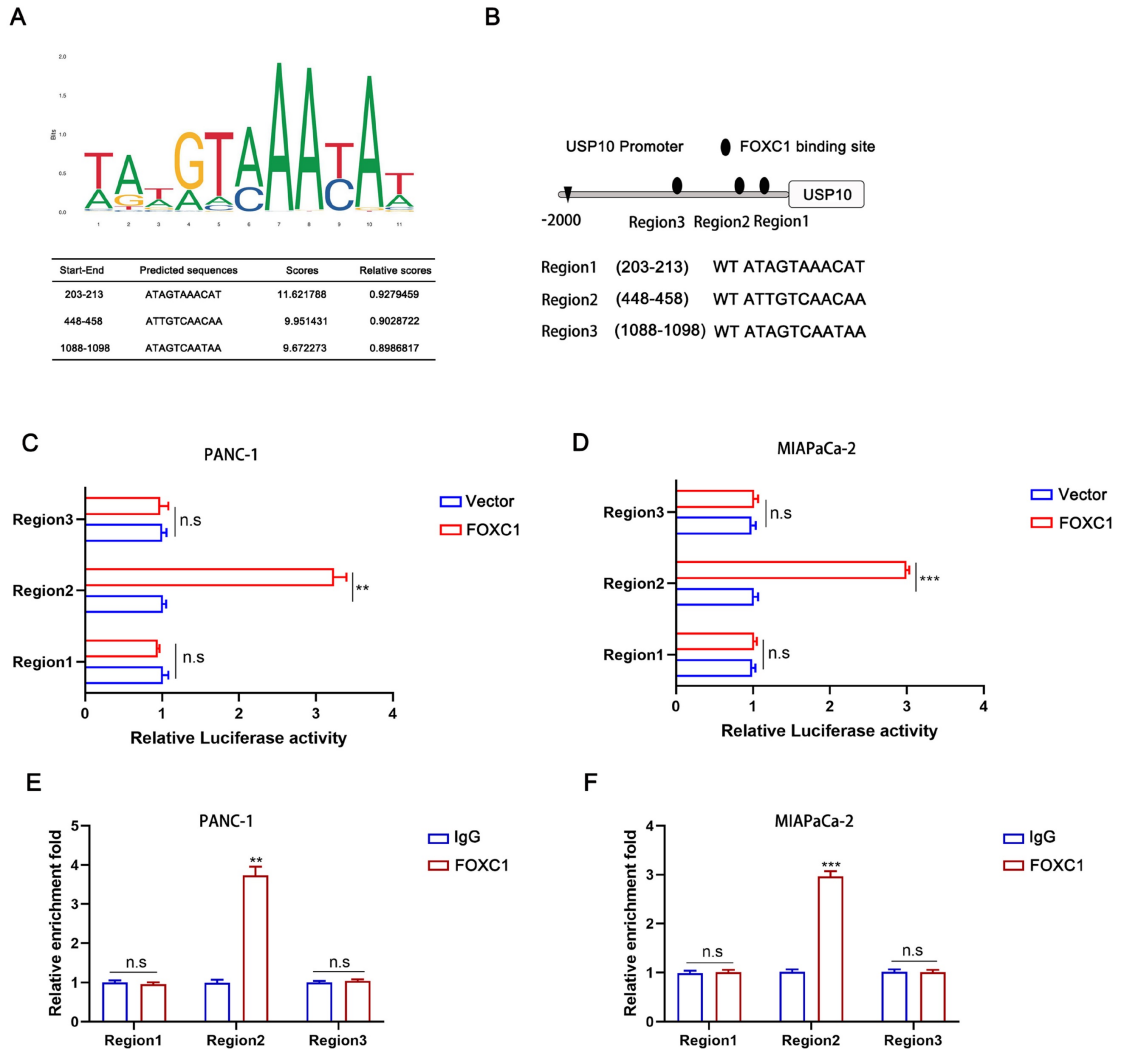
** USP10 can be transcriptional activated by FOXC1.** (a) The transcriptional binding sequences of FOXC1 and the promoter of USP10 based on JASPAR database. (b) The model diagram of promoter binding location and sequence. (c,d) The dual-luciferase reporter assays were performed to identify the exact binding region of USP10 promoter to FOXC1 in PANC-1 and MIA PaCa-2 cells. (e,f) Chip assays were further performed to identify the binding site of USP10 promoter to FOXC1 in PANC-1 and MIA PaCa-2 cells.

To investigate the underlying mechanism of how USP10 mRNA is upregulated by FOXC1 overexpression, we first screened the USP10 gene promoter region for possible FOXC1 binding sites using the JASPAR database (http://jaspardev.genereg.net/) (Figure 8A). We found three potential FOXC1 binding sites in the USP10 promoter, namely region 1 (203-213), region 2 (448-458), and region 3 (1088-1098) (Figure 8B). Dual-luciferase reporter assays were performed to further examine this, with the results suggesting that region 2 exhibited high FOXC1 binding. However, regions 1 and 3 had little promoter inducibility (Figure 8C/D). We then conducted ChIP assays, which also demonstrated that region 2 was the binding site for FOXC1 in the USP10 promoter at the chromatin level (Figure 8E/F). Taken together, FOXC1 can regulate USP10 at the transcriptional level to form a positive feedback control loop.

### FOXC1 reverses the anti-tumor effects of USP10 knockdown

**Figure 9:**
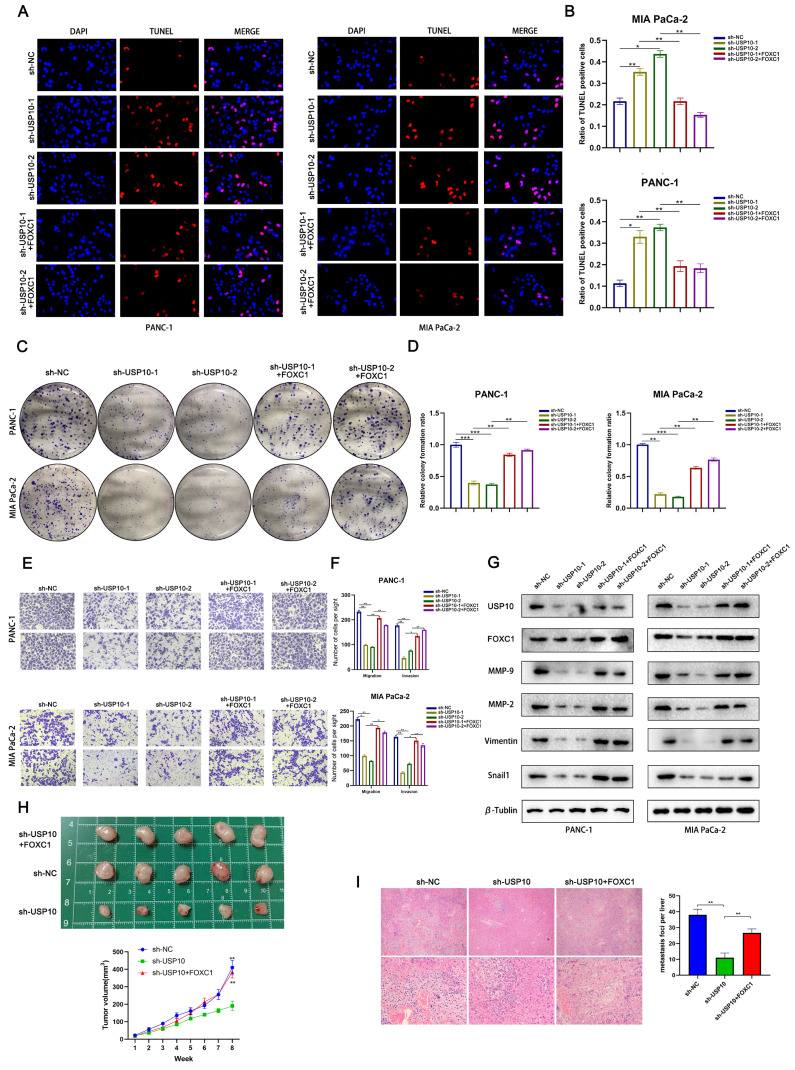
** FOXC1 reverses the anti-tumor effect of USP10 knockdown.** (a,b) TUNEL analysis were performed to clarify the apoptosis levels of PDAC in sh-NC group, sh-USP10 groups and sh-USP10+FOXC1 groups. (c,d) Colony forming assays were performed to proliferation abilities of PDAC in sh-NC group, sh-USP10 groups and sh-USP10+FOXC1 groups. (e,f) Transwell assays were performed to migration and invasion abilities of PDAC in sh-NC group, sh-USP10 groups and sh-USP10+FOXC1 groups. (g) WB analysis was performed to measure the levels of MMP-2. MMP-9. Vimentin and Snail1 in sh-NC group, sh-USP10 groups and sh-USP10+FOXC1 groups. (h) The 6-weeks old female BALB/c-nude mice were randomly divided into three groups (n=5) and subcutaneously inoculated with sh-NC, sh-USP10 or sh-USP10+FOXC1 PANC-1 cells (5×106 cells/100 µl) to established the xenograft PDAC bearing model. The photograph and the tumor volume curve of tumors sh-NC, sh-USP10 and sh-USP10+FOXC1 groups. (i) The 6-weeks old female BALB/c-nude mice were randomly divided into three groups (n=5) which were respectively injected with lentivirus contain sh-NC, sh-USP10 and sh-USP10+FOXC1 sequences to establish metastasis model. The livers of mice were dissected and measured by section observation under microscopy.

Our earlier results suggested that USP10 knockdown can significantly suppress PDAC cell proliferation and metastasis. To validate that the USP10-mediated tumor-promoting effects were dependent on FOXC1, we conducted a series of functional experiments. TUNEL assay data indicated that FOXC1 overexpression could partially abolish the USP10 knockdown-mediated pro-apoptotic effect (Figure 9A/B). In addition, we found that FOXC1 overexpression could partially reverse the inhibition of PDAC cell proliferation by USP10 knockdown (Figure 9C/D). Consistent with the colony formation assay results, our Transwell assay data also illustrated that FOXC1 overexpression could abolish the inhibition of PDAC cell migration and invasion by USP10 knockdown (Figure 9E/F). Western blot analysis suggested that the USP10 knockdown-mediated low expression patterns of MMP-2, MMP-9, Vimentin and Snail1 were abolished by FOXC1 overexpression (Figure 9G). Additionally, we performed animal experiments to verify if FOXC1 overexpression alone could restore cell proliferation and migration inhibition from USP10 knockdown. The results demonstrated that FOXC1 overexpression could restore both USP10-mediated cell proliferation and migration *in vivo* (Figure 9H/I). Overall, these results confirmed that the USP10-FOXC1 axis can promote PDAC cell proliferation and metastasis.

## Discussion

Chemotherapy and surgery are the main PDAC treatment options. However, only 15% to 20% of patients are eligible for surgery at the time of diagnosis. Most PDAC patients have distant metastases at diagnosis, and removal of the primary lesion by major surgery is unlikely to affect prognosis 22. Unfortunately, even in cases where surgical removal is possible, nearly 75% of patients relapse over two years, suggesting that micrometastatic disease was also present in these patients 23. Consequently, there is an urgent need to uncover the metastatic mechanism of PDAC. Evidence has suggested that dysfunctional protein expression is associated with PDAC malignant progression. Proteins, especially those in eukaryotic cells, maintain normal cellular function under homeostatic conditions, 80% of which are mediated by the ubiquitin-proteasome system 6. The dynamic regulation of ubiquitination and deubiquitination maintains the stability, activity, and localization of key proteins 24. Thus, when certain key metastasis-related proteins are modified by ubiquitination or deubiquitination, the carcinogenic effect may be amplified, resulting in the spread of PDAC.

In recent years, mounting data have indicated that DUBs play crucial roles in tumorigenesis 25. In the current study, we initiated a functional screening of a USP-targeting siRNA library for PDAC cell migration, finding that USP10 serves as a key regulator of FOCX1 expression to support WNT signaling pathway signaling and thereby ultimately increasing PDAC cell proliferation and metastasis. In addition, a WNT pathway inhibitor could partially inhibit the effects of FOXC1 on PDAC cells. Mechanistically, USP10 could directly interact with FOXC1 and promote its protein stability by deubiquitination, leading to FOXC1 overexpression. FOXC1 could activate the WNT signaling pathway to promote PDAC progression, while upregulating USP10 mRNA expression levels and forming a positive feedback loop. These results demonstrated that targeting USP10 is a potential novel therapeutic strategy for preventing PDAC metastasis.

Previous studies have suggested that USP10 is involved in various biological processes, including DNA repair, cell cycle regulation, autophagy, and tumorigenesis 26, 27. Li *et al.* reported that USP10 can participate in modulating the stability and expression levels of NLRP7 protein, which promoted the polarization of pro-tumor M2-like macrophages by inducing the secretion of C-C motif chemokine ligand 2. Downstream, NLPR7 could promote NF-κB signaling activation and facilitate colorectal cancer progression 28. Shi *et al.* suggested that USP10 could promoted IGF2BP1 protein stabilization by deubiquitination, resulting in high IGF2BP1 expression levels in breast cancer. IGF2BP1 could recognize and bind to the m6A sites on CPT1A mRNA, promoting the N6-methyladenosine modification and breast cancer metastasis 29. In PDAC studies, silencing USP10 expression led to an unfolded protein response and upregulation of PERK and IRE1α, resulting in increased endoplasmic reticulum stress that thus ultimately decreased the metastatic potential 11. However, a more detailed metastatic mechanism of PDAC was unclear. In this study, we focused on a new binding protein, FOXC1, which is also a reported oncogene in many tumor types. Our mass spectrometry analysis results indicated that USP10 potentially interacts with SIRT6, p53, and RAF1. Previous studies have suggested that USP10 can promote Raf-1 protein expression and activate the Raf-1/MEK/ERK pathway in endometriosis and glioblastoma. USP10 inhibits hepatic steatosis, insulin resistance, and inflammation through Sirt6. Additionally, USP10 functions as a Sirt6 deubiquitinase to promote tumorigenesis and cardiac myocyte hypertrophy. Furthermore, p53 protein stability and localization can be regulated by USP10, with USP10 suppressing tumor cell growth in cells with wild-type p53. The aforementioned studies demonstrate the protein interactions between USP10 and SIRT6, p53, and RAF1. However, direct evidence supporting their involvement in PDAC is lacking. Nevertheless, it is undeniable that USP10 may contribute to PDAC progression through its interactions with these proteins. Considering these findings, the current study focused on investigating FOXC1, which has not been previously studied, to elucidate its role in conjunction with USP10 in PDAC. Therefore, we found that knocking down USP10 expression could mediate anti-tumor effects, which were partially blocked by FOXC1. Additionally, FOXC1 overexpression was regulated by USP10 via deubiquitination. These finding suggested that USP10 could function as an oncogene to precisely control PDAC progression by stabilizing FOXC1 protein.

FOXC1 is a key regulator of lineage norms and its expression is highly regulated throughout development. Phosphorylation of S272 is key for regulating FOXC1 stability and transcriptional activity 19, 20. ERK1/2-mediated phosphorylation of S272 inhibits rapid FOXC1 degradation by the 26S proteasome. Interestingly, we found that phosphorylation of S272 depends on the deubiquitination modification of FOXC1 by USP10, which stabilized FOXC1 localization in the nucleus and decreased its degradation.

Numerous studies have confirmed that FOXC1 overexpression is frequently observed in cancers, with reports in more than 16 types of cancer, and is related to unfavorable prognosis. Because FOXC1 is a transcription factor and central hub gene that controls hundreds of gene networks, upregulated FOXC1 expression in cancer has widespread effects on many key biological processes related to tumor growth. During cancer progression and metastasis, FOXC1 mediates the cellular plasticity, partial EMT, treatment resistance, invasion, and migration of tumor stem cells 30. In addition, FOXC1 is interconnected with multiple signaling pathway, such as EGFR, NF-κB, ERK, and PI3K/AKT 31-33. Here, we found that FOCX1 knockdown inhibited PDAC cell proliferation, migration, and invasion. By integrating sequencing, bioinformatics predictions, and other experimental data, we determined the underlying mechanism to be that USP10 can induce FOXC1 overexpression and activate the WNT signaling pathway. A previous study in gastric cancer confirmed that FOXC1 binds to the promoter region of the β-catenin gene and transactivates its expression, which activates WNT signaling 34. Another study found that FOXC1 can also form complexes with the unphosphorylated β-catenin protein in the cytoplasm, thereby facilitating β-catenin entry into the nucleus. Once in the nucleus, FOXC1 separates from β-catenin, thereby regulating c-MYC transcription and promoting gastric cancer cell proliferation 35. Luciferase reporter and ChIP assay data from Cao *et al.* indicated that β-catenin can be a direct transcriptional target of FOXC1. FOXC1 promotes cancer stem cell-like properties by upregulating β-catenin and activating WNT signaling 36. Taken together, our findings suggested that FOXC1 can activate WNT signaling by regulating β-catenin transcription. Thus, USP10 can upregulate FOXC1 expression and activate WNT signaling, which promotes PDAC cell proliferation and metastasis. Interestingly, we also found that FOXC1 could bind to the USP10 gene promoter, supporting USP10 transcriptional activation. These data indicate the presence of a positive feedback loop between USP10 and FOXC1 that can further support PDAC malignant progression.

## Conclusion

In summary, our study demonstrated that USP10 is a key mediator of PDAC progression through supporting FOXC1 protein stability via deubiquitination. FOXC1 can promote USP10 transcriptional activity, suggesting that USP10 and FOXC1 form a positive feedback loop to activate WNT signaling and accelerate PDAC progression. USP10 represents an exciting therapeutic target that is a potential new strategy for treating PDAC.

## Supplementary Material

Supplementary figures and tables.

## Figures and Tables

**Figure 10:**
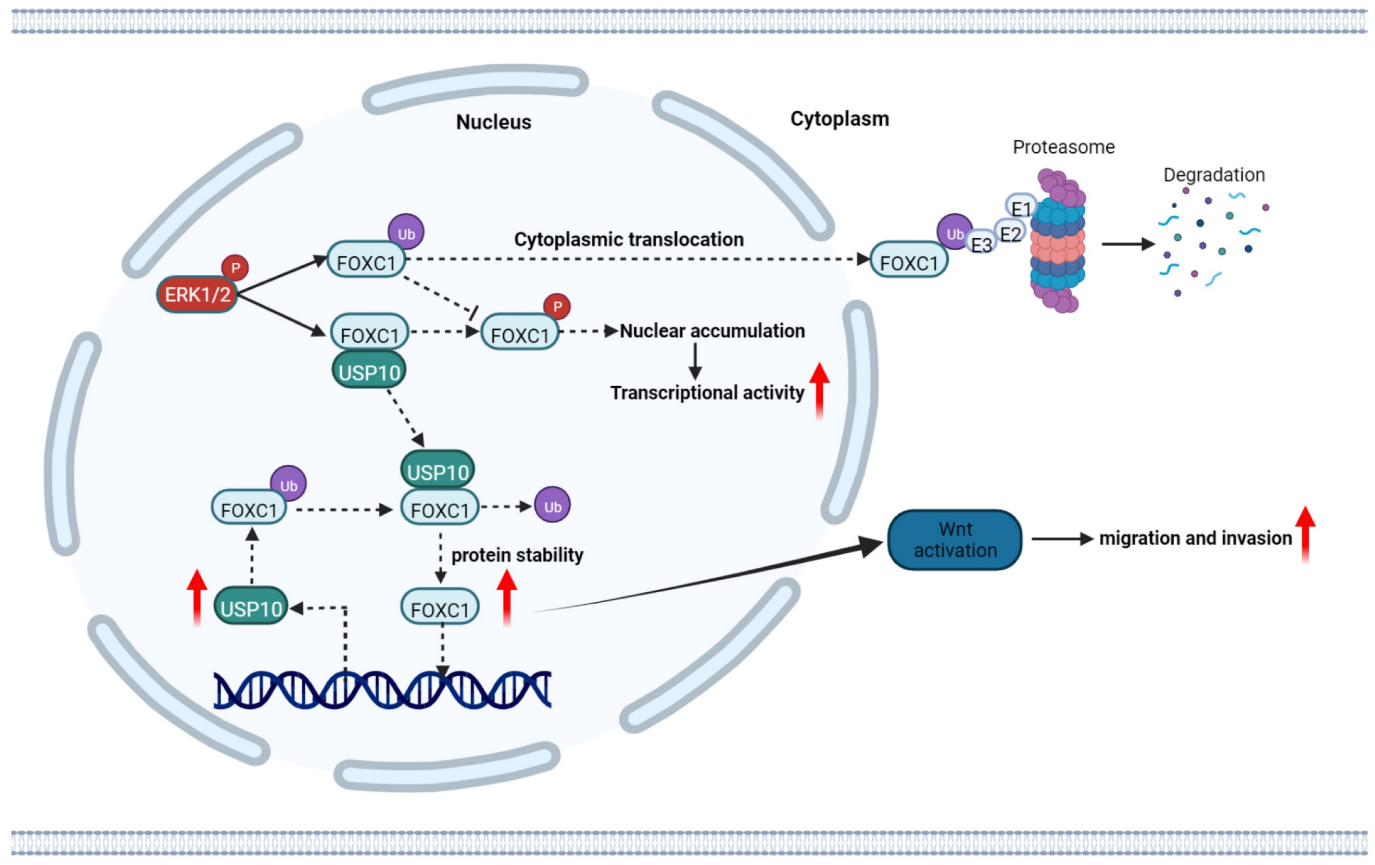
** The schematic diagram of USP10 promoting pancreatic cancer progression.** USP10 is a key mediator of PDAC maintenance and survival through promoting FOXC1 protein stability by deubiquitination, whereas FOXC1 promoted USP10 transcriptional activity. USP10 and FOXC1 form a positively feedback loop to activate WNT signaling and accelerate PDAC progression.

**Table 1:** Association of USP10 expression with clinical case data of PDAC patients

Clinicopathologic Feature		USP10		*p value*
		High expression	Low expression	
All cases		24	21	
Age				
	≤55	11	14	0.2312
	>55	13	7	
Gender				
	male	14	8	0.2362
	female	10	13	
Diameter of tumor				
	≤2	7	10	0.2333
	>2	17	11	
TNM stage				
	Ⅰ/Ⅱ	16	15	0.7588
	Ⅲ/Ⅳ	8	6	
Lymphatic metastasis				
	Negative	7	14	*0.0174*
	Positive	17	7	
Distant metastasis				
	Negative	20	19	0.6695
	Positive	4	2	
Pathological grading				
	Ⅰ/Ⅱ	14	15	0.5334
	Ⅲ	10	6	

Italics bold indicates p is less than 0.05.

## Abbreviations

PDAC: pancreatic ductal adenocarcinoma
DUB: deubiquitinating enzyme
USP: ubiquitin-specific protease
Forkhead box C1: FOXC1
Co-IP: Co-immunoprecipitation
FBS: fetal bovine serum
HPDE: human pancreatic ductal epithelial cell
RT-qPCR: Real-time fluorescence quantitative PCR
GSEA: gene set enrichment analysis
GO: gene ontology
KEGG: kyoto encyclopedia of genes and genomes
OS: overall survival
DFS: disease free survival
TCGA: The Cancer Genome Atlas

## References

[1] Qin C, Wang Y, Zhao B, Li Z, Li T, Yang X (2023). STOML2 restricts mitophagy and increases chemosensitivity in pancreatic cancer through stabilizing PARL-induced PINK1 degradation. Cell Death Dis.

[2] Siegel RL, Miller KD, Fuchs HE, Jemal A (2022). Cancer statistics, 2022. Ca-Cancer J Clin.

[3] Cai K, Chen S, Zhu C, Li L, Yu C, He Z (2022). FOXD1 facilitates pancreatic cancer cell proliferation, invasion, and metastasis by regulating GLUT1-mediated aerobic glycolysis. Cell Death Dis.

[4] Halbrook CJ, Lyssiotis CA, Pasca DMM, Maitra A (2023). Pancreatic cancer: Advances and challenges. Cell.

[5] Nimmakayala RK, Ogunleye AO, Parte S, Krishna KN, Raut P, Varadharaj V (2022). PAF1 cooperates with YAP1 in metaplastic ducts to promote pancreatic cancer. Cell Death Dis.

[6] Tao L, Liu X, Jiang X, Zhang K, Wang Y, Li X (2022). USP10 as a Potential Therapeutic Target in Human Cancers. Genes (Basel).

[7] Chen R, Pang X, Li L, Zeng Z, Chen M, Zhang S (2022). Ubiquitin-specific proteases in inflammatory bowel disease-related signalling pathway regulation. Cell Death Dis.

[8] Hou P, Ma X, Zhang Q, Wu CJ, Liao W, Li J (2019). USP21 deubiquitinase promotes pancreas cancer cell stemness via Wnt pathway activation. Gene Dev.

[9] Harrigan JA, Jacq X, Martin NM, Jackson SP (2018). Deubiquitylating enzymes and drug discovery: emerging opportunities. Nat Rev Drug Discov.

[10] Nelson JK, Thin MZ, Evan T, Howell S, Wu M, Almeida B (2022). USP25 promotes pathological HIF-1-driven metabolic reprogramming and is a potential therapeutic target in pancreatic cancer. Nat Commun.

[11] Bhattacharya U, Thavathiru E, Neizer-Ashun F, Xu C, Gatalica Z, Dwivedi S (2022). The deubiquitinase USP10 protects pancreatic cancer cells from endoplasmic reticulum stress. Npj Precis Oncol.

[12] Quan G, Xu J, Wang J, Liu X, Xu J, Jiang J (2023). KIF15 is essential for USP10-mediated PGK1 deubiquitination during the glycolysis of pancreatic cancer. Cell Death Dis.

[13] Han B, Bhowmick N, Qu Y, Chung S, Giuliano AE, Cui X (2017). FOXC1: an emerging marker and therapeutic target for cancer. Oncogene.

[14] Gilding LN, Somervaille T (2019). The Diverse Consequences of FOXC1 Deregulation in Cancer. Cancers.

[15] Subramani R, Camacho FA, Levin CI, Flores K, Clift A, Galvez A (2018). FOXC1 plays a crucial role in the growth of pancreatic cancer. Oncogenesis.

[16] Zhao L, Liu Y, Zhang J, Liu Y, Qi Q (2019). LncRNA SNHG14/miR-5590-3p/ZEB1 positive feedback loop promoted diffuse large B cell lymphoma progression and immune evasion through regulating PD-1/PD-L1 checkpoint. Cell Death Dis.

[17] Lange SM, Armstrong LA, Kulathu Y (2022). Deubiquitinases: From mechanisms to their inhibition by small molecules. Mol Cell.

[18] Stalnecker CA, Grover KR, Edwards AC, Coleman MF, Yang R, DeLiberty JM (2022). Concurrent Inhibition of IGF1R and ERK Increases Pancreatic Cancer Sensitivity to Autophagy Inhibitors. Cancer Res.

[19] Berry FB, Mirzayans F, Walter MA (2006). Regulation of FOXC1 stability and transcriptional activity by an epidermal growth factor-activated mitogen-activated protein kinase signaling cascade. J Biol Chem.

[20] Zhang Y, Liao Y, Chen C, Sun W, Sun X, Liu Y (2020). p38-regulated FOXC1 stability is required for colorectal cancer metastasis. J Pathol.

[21] Nahm JH, Yang WI, Yoon SO (2020). Forkhead Box C1 (FOXC1) Expression in Stromal Cells within the Microenvironment of T and NK Cell Lymphomas: Association with Tumor Dormancy and Activation. Cancer Res Treat.

[22] Cronin KA, Scott S, Firth AU, Sung H, Henley SJ, Sherman RL (2022). Annual report to the nation on the status of cancer, part 1: National cancer statistics. Cancer-Am Cancer Soc.

[23] Groot VP, Rezaee N, Wu W, Cameron JL, Fishman EK, Hruban RH (2018). Patterns, Timing, and Predictors of Recurrence Following Pancreatectomy for Pancreatic Ductal Adenocarcinoma. Ann Surg.

[24] Aliabadi F, Sohrabi B, Mostafavi E, Pazoki-Toroudi H, Webster TJ (2021). Ubiquitin-proteasome system and the role of its inhibitors in cancer therapy. Open Biol.

[25] Bhattacharya U, Neizer-Ashun F, Mukherjee P, Bhattacharya R (2020). When the chains do not break: the role of USP10 in physiology and pathology. Cell Death Dis.

[26] Yang J, Meng C, Weisberg E, Case A, Lamberto I, Magin RS (2020). Inhibition of the deubiquitinase USP10 induces degradation of SYK. Brit J Cancer.

[27] Li S, Zhu Y, Zhang T, Hang Y, Chen Q, Jin Y (2019). Cai's Neiyi Prescription promotes apoptosis and inhibits inflammation in endometrial stromal cells with endometriosis through inhibiting USP10. Biotechnol Appl Bioc.

[28] Li B, Qi ZP, He DL, Chen ZH, Liu JY, Wong MW (2021). NLRP7 deubiquitination by USP10 promotes tumor progression and tumor-associated macrophage polarization in colorectal cancer. J Exp Clin Canc Res.

[29] Shi J, Zhang Q, Yin X, Ye J, Gao S, Chen C (2023). Stabilization of IGF2BP1 by USP10 promotes breast cancer metastasis via CPT1A in an m6A-dependent manner. Int J Biol Sci.

[30] Ray T, Ryusaki T, Ray PS (2021). Therapeutically Targeting Cancers That Overexpress FOXC1: A Transcriptional Driver of Cell Plasticity, Partial EMT, and Cancer Metastasis. Front Oncol.

[31] Jin Y, Han B, Chen J, Wiedemeyer R, Orsulic S, Bose S (2014). FOXC1 is a critical mediator of EGFR function in human basal-like breast cancer. Ann Surg Oncol.

[32] Chung S, Jin Y, Han B, Qu Y, Gao B, Giuliano AE (2017). Identification of EGF-NF-kappaB-FOXC1 signaling axis in basal-like breast cancer. Cell Commun Signal.

[33] Nguyen K, Yan Y, Yuan B, Dasgupta A, Sun J, Mu H (2018). ST8SIA1 Regulates Tumor Growth and Metastasis in TNBC by Activating the FAK-AKT-mTOR Signaling Pathway. Mol Cancer Ther.

[34] Sun Y, Lin C, Ding Q, Dai Y (2022). Overexpression of FOXC1 Promotes Tumor Metastasis by Activating the Wnt/beta-Catenin Signaling Pathway in Gastric Cancer. Digest Dis Sci.

[35] Jiang J, Li J, Yao W, Wang W, Shi B, Yuan F (2021). FOXC1 Negatively Regulates DKK1 Expression to Promote Gastric Cancer Cell Proliferation Through Activation of Wnt Signaling Pathway. Front Cell Dev Biol.

[36] Cao S, Wang Z, Gao X, He W, Cai Y, Chen H (2018). FOXC1 induces cancer stem cell-like properties through upregulation of beta-catenin in NSCLC. J Exp Clin Canc Res.

