# Supplementary material for: USP10 promotes pancreatic ductal adenocarcinoma progression by attenuating FOXC1 protein degradation to activate the WNT signaling pathway
Source: Int J Biol Sci. 2024 Sep 30;20(13):5343–62. doi: 10.7150/ijbs.92278 (PMC11488585; doi:10.7150/ijbs.92278)
Supplement: Supplementary file 1 — Supplementary figures and tables. [file ijbsv20p5343s1.pdf]

Supplemental Figure 1. USP10 overexpression promoted the protein expression of FOXC1 . (a) PCR analysis was perform to measure the effect of USP10 overexpression on the mRNA of FOXC1.(b) Western blot was perform to measure the effect of USP10 overexpression on the protein of FOXC1.

Supplemental Figure 2. FOXC1 was upregulated in PDAC and high FOXC1 expression was remarkably associated with poor prognosis. (a) The expression of FOXC1 in PDAC tissues based on TCGA database. (b) The overall survival rate of the PDAC patients with high or low FOXC1 expression was estimated by Kaplan-Meier analysis. (c) The disease free survival rate of the PDAC patients with high or low FOXC1 expression was estimated by Kaplan-Meier analysis. (d) The expression of FOXC1 was analyzed in PDAC cell lines by using PCR analysis. (e) The expression of FOXC1 was analyzed in PDAC cell lines by using WB analysis. (f) The expression of FOXC1 was analyzed in PDAC tissues by using WB analysis. (g) The transfection effects of sh-FOXC1 were evaluated by PCR analysis. (h) The effect of FOXC1 on cell proliferation in PDAC cells were measured by colony forming assays. (i) The effect of FOXC1 on cell apoptosis in PDAC cells were measured by Tunel assays.

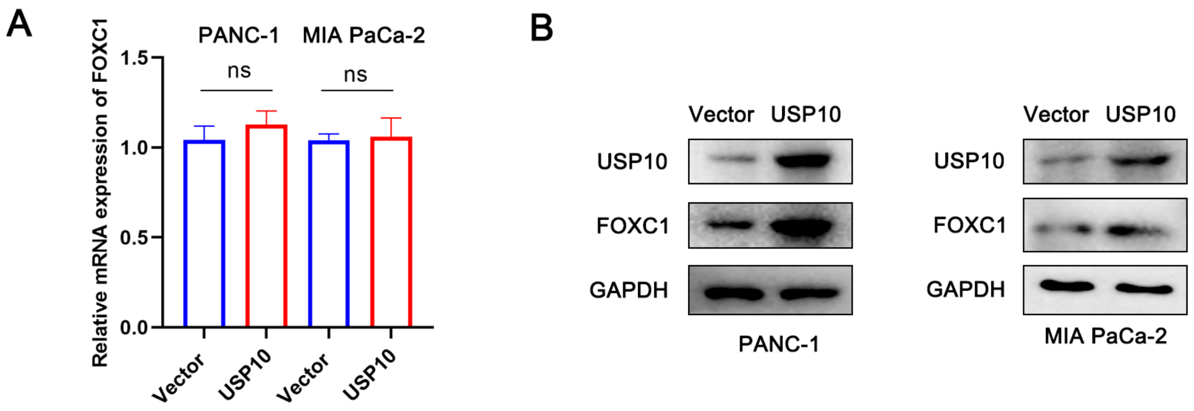
A
PANC-1
MIA PaCa-2
ns
ns
Relative mRNA expression of FOXC1
1.5
1.0
0.5
0.0
Vector
USP10
Vector
USP10
B
Vector
USP10
USP10
FOXC1
GAPDH
PANC-1
Vector
USP10
USP10
FOXC1
GAPDH
MIA PaCa-2

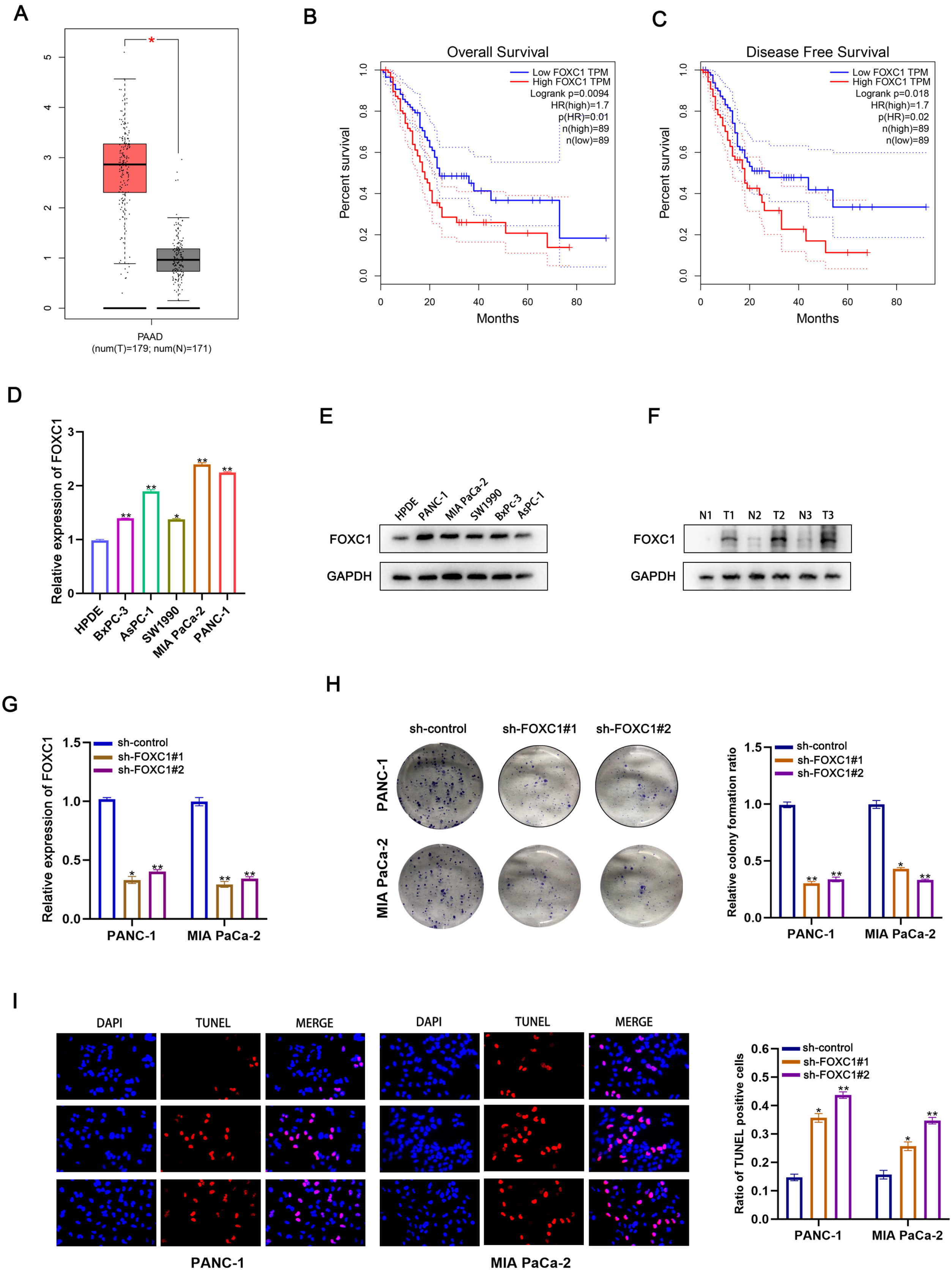
A
PAAD
(num(T)=179; num(N)=171)
B
Overall Survival
Low FOXC1 TPM
High FOXC1 TPM
Logrank p=0.0094
HR(high)=1.7
p(HR)=0.01
n(high)=89
n(low)=89
Percent survival
Months
C
Disease Free Survival
Low FOXC1 TPM
High FOXC1 TPM
Logrank p=0.018
HR(high)=1.7
p(HR)=0.02
n(high)=89
n(low)=89
Percent survival
Months
D
Relative expression of FOXC1
HPDE
BxPC-3
AsPC-1
SW1990
MIA PaCa-2
PANC-1
E
HPDE
PANC-1
MIA PaCa-2
SW1990
BxPC-3
AsPC-1
FOXC1
GAPDH
F
N1
T1
N2
T2
N3
T3
FOXC1
GAPDH
G
sh-control
sh-FOXC1#1
sh-FOXC1#2
Relative expression of FOXC1
PANC-1
MIA PaCa-2
H
sh-control
sh-FOXC1#1
sh-FOXC1#2
PANC-1
MIA PaCa-2
Relative colony formation ratio
I
DAPI
TUNEL
MERGE
PANC-1
MIA PaCa-2
Ratio of TUNEL positive cells

Supplemental Table 1. The sequences involved in this study.

Gene		Sequence (5'—3')
USP10	Forward	AAGCAAGCTATGGCTCCATCG
	Reverse	CTTCCGCCTCCACATTAGAAC
FOXC1	Forward	TGTTCGAGTCACAGAGGATCG
	Reverse	ACAGTCGTAGACGAAAGCTCC
GAPDH	Forward	CTGGGCTACACTGAGCACC
	Reverse	AAGTGGTCGTTGAGGGCAATG

Supplementary Table 2. The sequences of shRNA in this study.

Name	Sequences:5'→3'
sh-USP10-1	CCGGGGACAAGAAUAUCAGAGAAUUCTCGAGUUCUCUGAUAUUCUUGUCCAUTTTTTG
sh-USP10-2	CCGGGCAGGUUGAAGUCAAAGAAGGCTCGAGUUCUUUGACUUCAACCUGCUUTTTTTG
sh-NC	CCGGCAACAAGATGAAGAGCACCAACTCGAGTTGGTGCTCTTCATCTTGTTGTTTTTG
sh-FOXC1-1	CCGGCGGGAAUAGUAGCUGUCAAAUCTCGAGUUGACAGCUACUAUUCCCGUUTTTTTG
sh-FOXC1-2	CCGGCCUACAACAUGUUCGAGAACGCTCGAGUUCUCGAACAUGUUGUAGGAGTTTTTG
sh-control	CCGGCAACAAGATGAAGAGCACCAACTCGAGTTGGTGCTCTTCATCTTGTTGTTTTTG
